# Patients with a severe prolonged Disorder of Consciousness can show classical EEG responses to their own name compared with others' names

**DOI:** 10.1016/j.nicl.2018.04.027

**Published:** 2018-04-30

**Authors:** Agnieszka M. Kempny, Leon James, Kudret Yelden, Sophie Duport, Simon F. Farmer, E. Diane Playford, Alexander P. Leff

**Affiliations:** a The Institute of Neuro-palliative Rehabilitation, Royal Hospital for Neuro-disability, London SW15 3SW, UK; b Department of Brain Repair and Rehabilitation, Institute of Neurology, University College London, Queen Square, London WC1N 3BG, UK; c Institute of Cognitive Neuroscience, University College London, Queen Square, WC1N 3AR London, UK; d The National Hospital for Neurology & Neurosurgery, Queen Square, London WC1N 3BG, UK; e Health Sciences, Warwick Medical School, University of Warwick, Coventry CV4 7AL, UK

## Abstract

Patients in Vegetative State (VS), also known as Unresponsive Wakefulness State (UWS) are deemed to be unaware of themselves or their environment. This is different from patients diagnosed with Minimally Conscious state (MCS), who can have intermittent awareness. In both states, there is a severe impairment of consciousness; these disorders are referred to as disorders of consciousness (DOC) and if the state is prolonged, pDOC. There is growing evidence that some patients who are behaviourally in VS/UWS can show neural activation to environmental stimuli and that this response can be detected using functional brain imaging (fMRI/PET) and electroencephalography (EEG). Recently, it has also been suggested that a more reliable detection of brain responsiveness and hence a more reliable differentiation between VS/UWS and MCS requires person-centred and person-specific stimuli, such as the subject's own name stimulus.

In this study we obtained event related potential data (ERP) from 12 healthy subjects and 16 patients in pDOC, five of whom were in the VS/UWS and 11 in the Minimally Conscious State (MCS). We used as the ERP stimuli the subjects' own name, others' names and reversed other names. We performed a sensor level analysis using Statistical Parametric Mapping (SPM) software. Using this paradigm in 4 DOC patients (3 in MCS, and 1 in VS/UWS) we detected a statistically significant difference in EEG response to their own name versus other peoples' names with ERP latencies (~300 ms and ~700 ms post stimuli). Some of these differences were similar to those found in a control group of healthy subjects.

This study shows the feasibility of using self-relevant stimuli such as a subject's own name for assessment of brain function in pDOC patients. This neurophysiological test is suitable for bed-side/hospital based assessment of pDOC patients. As it does not require sophisticated scanning equipment it can feasibly be used within a hospital or care setting to help professionals tailor medical and psycho-social management for patients.

## Highlights

• Approximately 25% of patients in disorders of consciousness retain ability to respond to own first name using electroencephalography
• Timing and topography of these responses is in keeping with control subjects data
• 3 The use semantically salient stimuli, such as one's own name may give additional information when assessing brain function in pDOC patients

## 1 Introduction

Previously, patients who emerged from coma following a severe brain injury, into a state of wakefulness but unresponsiveness were diagnosed as being in a Vegetative State (VS) (Jennett and Plum, 1972), also called the Unresponsive Wakefulness Syndrome (UWS) (Laureys et al., 2010). Later it became apparent that this group of patients is heterogeneous and that some patients demonstrate self-awareness and environmental awareness, albeit in an inconsistent manner. Hence, a new diagnostic term of Minimally Conscious State (MCS) was introduced by Giacino et al. (Giacino et al., 2002) in order to distinguish this patient group form those in the VS/UWS state. Both types of patients (UWS and MCS) are now said to suffer from a Disorder of Consciousness (DOC) or prolonged Disorder of Consciousness (pDOC), which recognizes that the two sub-types exist on a continuum (Turner-Stokes, 2014). Furthermore, some VS/UWS patients defined by behavioural criteria can show a degree of awareness, and could be thought of as being in a “functional locked-in syndrome” rather than in VS or UWS (Coleman et al., 2007; Coleman et al., 2009) (Owen et al., 2006; Monti et al., 2010; Boly et al., 2011; Formisano et al., 2013). It has been estimated that approximately 10% of the patients with a diagnosis of VS/UWS, have some preserved higher brain function (Turner-Stokes et al., 2012).

The methods for detecting unexpected levels of self-awareness have relied on technological approaches such as functional Magnetic Resonance Imaging (fMRI) or electroencephalography (EEG), rather than clinical rating scores that rely on observations of the patient's externally expressed behaviour (Coleman et al., 2009; Cruse et al., 2011). A particularly salient auditory stimulus is the subject's own name (SON) (Moray, 1959) and it has been suggested that this could be used as a neurophysiological approach for detecting higher-level brain function in pDOC patients (Cavinato et al., 2011; Cruse et al., 2014), (Risetti et al., 2013). The EEG brain responses to a SON are assessed using event related potentials (ERP). ERP responses to SON auditory stimuli in healthy adults' subjects have two main components that occur around 250 ms and 800 ms post stimulus; these are called the early and late response, respectively. These are of positive polarity and are widely distributed over the frontal and parietal areas (Polich, 2007; Hauger et al., 2015). Both responses are considered to represent cognitive potentials. The early response is deemed to reflect cognitive functions such as stimulus recognition and working memory updating (Coles et al., 1988), (Polich, 1987), while the late response is believed to occur during recollection and retrieval processes (Holeckova et al., 2006). Researchers have used a variety of different paradigms to elicit name-specific ERPs. More passive paradigms (such as the one we utilize) have been shown to identify greater responses to others' names than own name in control participants, particularly affecting the P300 component (Hauger et al., 2015).

The aim of our study was to assess the brain responses to a SON paradigm in patients with pDOC to see if we could identify subjects, at either the group or single-subject level, who had consistent ERP responses in time and neuroanatomical space resembling those of healthy controls.

## 2 Methods

### 2.1 Subjects

#### 2.1.1 Controls

Twelve healthy, right-handed volunteers, five female, mean age of 39.09 years, (SD = 5.26) were included. None had suffered from neurological or psychiatric disorders or brain injury; none were taking psychotropic medications.

#### 2.1.2 Prolonged Disorders of Consciousness patients

We recruited 16 patients from the Royal Hospital for Neuro-disability in London, UK. This 26-bedded unit provides a comprehensive neuro-rehabilitation programme for patients with DOC in the post-acute phase following severe brain injury (GCS < 8).

The patient inclusion criteria were as follows: 1) severe acquired brain injury leading to prolonged DOC (longer than 4 weeks since brain injury) or permanent DOC. A permanent DOC was defined according to the Royal College of Physician Guidance (RCP, 2013), that is, lasting longer than 12 months if the aetiology is trauma, or longer than 6 months for anoxia and other causes; 2) conservative management of brain injury (no neurosurgery); 3) at least unilaterally intact brainstem auditory evoked potentials (BAEP).

All patients were assessed using The Sensory Modality Assessment and Rehabilitation Technique (SMART) (Gill-Thwaites and Munday, 2004). The SMART scale scores responses to sensory and environmental stimuli during ten one-to-one (patient-assessor) sessions lasting approximately 60 min each. In all patients, the diagnosis of VS/UWS or MCS remained stable on SMART testing throughout the assessment and ERP testing period.

Written informed consent was obtained from all the control subjects and an assent from the relatives of the patients prior to the study. The investigation was carried out in accordance with the latest version of the Declaration of Helsinki and the study was approved by a National Research Ethics Committee (NRES Committee London-Queen Square, REC reference number 11/LO/1233, SSA reference number 11/LO/2052).

### 2.2 Experimental paradigm

**Fig. 1:**
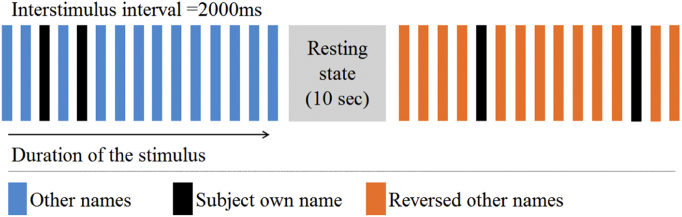
Auditory paradigm, each activation block lasted for 35.5 s, 15 stimuli per block with the random presentation of two SONs and 13 other stimuli or reversed names, in total 22 blocks were presented, 11 with the other names and 11 with reversed names.

During the experiment three types of auditory stimuli were presented: i) SON, ii) other names and iii) time-reversed other names. There were three block types: others' names, reversed names and rest (no auditory stimuli). Each block was started with an auditory cue, recorded by the same male voice as used for all names in this experiment “listen to the following names”. Two SON trials were randomly inserted in the two auditory blocks (others' and reversed names, see Fig. 1), so that they would be flanked by acoustically similar but semantically varying stimuli in the block. The auditory stimuli were presented in blocks totalling 15 stimuli each. The inter-stimulus interval was fixed at 2000 ms. Each auditory block lasted for 35.5 s and was followed by a 10 s long rest block. Twenty-two blocks were presented producing in total for each subject 44 SON trials, 143 others' name trials and 143 reversed name trials. The duration of the auditory stimuli ranged from 292 ms to 736 ms, average = 500 ms. During the recoding the patients remained awake and if case of drowsiness an activation protocol was used as described by Giacino (Giacino et al., 2004).

We focused our analyses on the contrast of own name vs. others' names because these two pairs of stimuli are the closest in terms of acoustic information. Others' names and own name contain both phonological and semantic information but mainly differ in terms of semantic salience (Arnell et al., 1999). While reversed names offer a good acoustic control (they are broadband stimuli) and contain some phonological elements of normal speech, they differ in both their phonological form (in particular phonotactics) as well as their lexico-semantic content (Leff et al., 2008).

The auditory stimuli (names) were recorded by a male native English speaker using a Magix music editor version 2.0 and the audio files were edited using the Praat software (free computer software developed by Paul Boersma and David Weenink, from the Institute of Phonetic Sciences - University of Amsterdam). The reversed name stimuli were simply time reversed other names used in this experiment (the spectrogram was reflected across the midpoint of the time axis). There were four different other name and time-reversed stimuli that were repeated 143 times. The stimuli were delivered binaurally through earphones using evoke software 3.1.5 (*ANT Neuro*, *Enscheda*, *The Netherlands*) with sound levels set to 70 dB.

### 2.3 EEG data registration and analysis

**Fig. 2:**
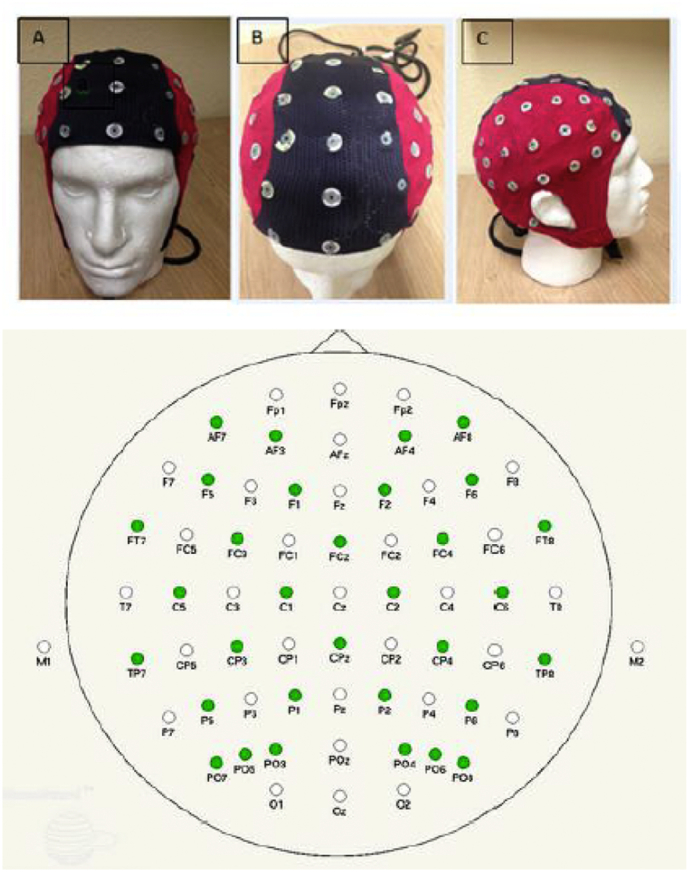
The EEG cap layout, the top figures shows frontal, top and lateral view of the cap, the bottom figure shows the 10–20 layout with an additional 10–10 electrodes, the layout provided by the ANT-Neuro, Enscheda, the Netherlands.

The EEG data were acquired using a Waveguard 64 EEG sensor-cap (*ANT-Neuro*, *Enscheda*, *the Netherlands*) (Fig. 2) whose standard sensor positions were derived from the 10–20 system (Jasper, 1958) with additional positions being determined from the 10–10 electrode placement system (Koessler et al., 2009). The EEG sensors were configured in a bipolar montage. The online filtering regime was 0.53–40 Hz, without mains suppression; data was sampled at 512 Hz. Online sensitivity was set to 70 μV/cm and a ground electrode was placed at FpZ. The electrode impedances were kept below 5 Kohm.

**Fig. 3:**
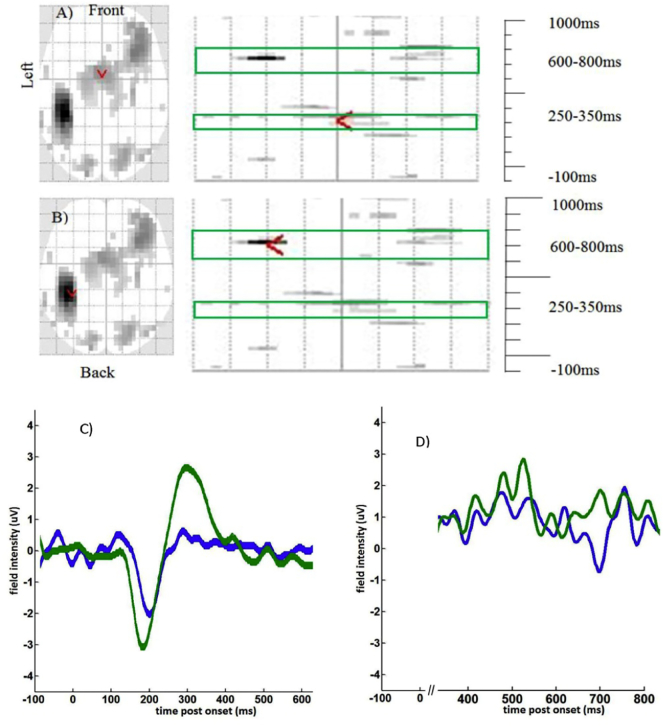
Results of F test difference between responses to own name and other names within the window of −100 ms to 1000 ms in per stimulus time. The red arrow indicates the most significant response from Healthy control subjects (*n* = 10), using an F test to contrast ‘own name’ against ‘others’ names' A) Early response 250–350 ms (midline frontal), B) Late response 600–800 ms, (left superior parietal), C) ERP response 250–350 ms to own name-blue line and other names-green line, D) ERP response 600–800 ms to own name-blue line and other names-green line.

**Fig. 4:**
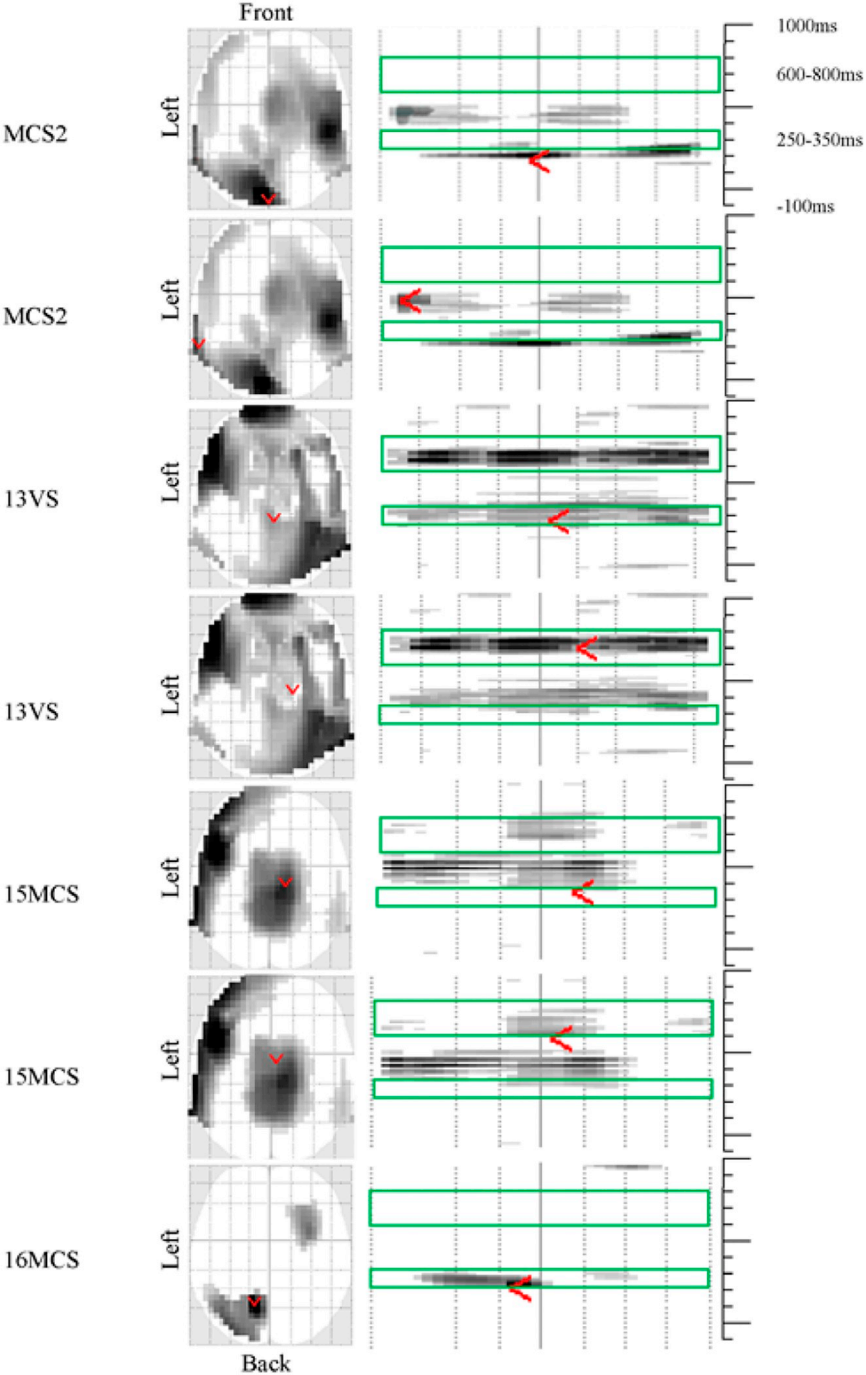
Results of F test difference between responses to own name and other names within the window of −100 ms to 1000 ms. Early (250–350 ms) response and late (600 − 800 ms) response in four pDOC patients showing the F test results with the statistical significant differences between responses to own name vs. responses to other names. The red arrow indicates the most significant response indicating the spatial position of the most significant responses. VS/UWS- Vegetative State/Unresponsive Wakefulness Stata, MCS- Minimally Conscious State.

**Fig. 5:**
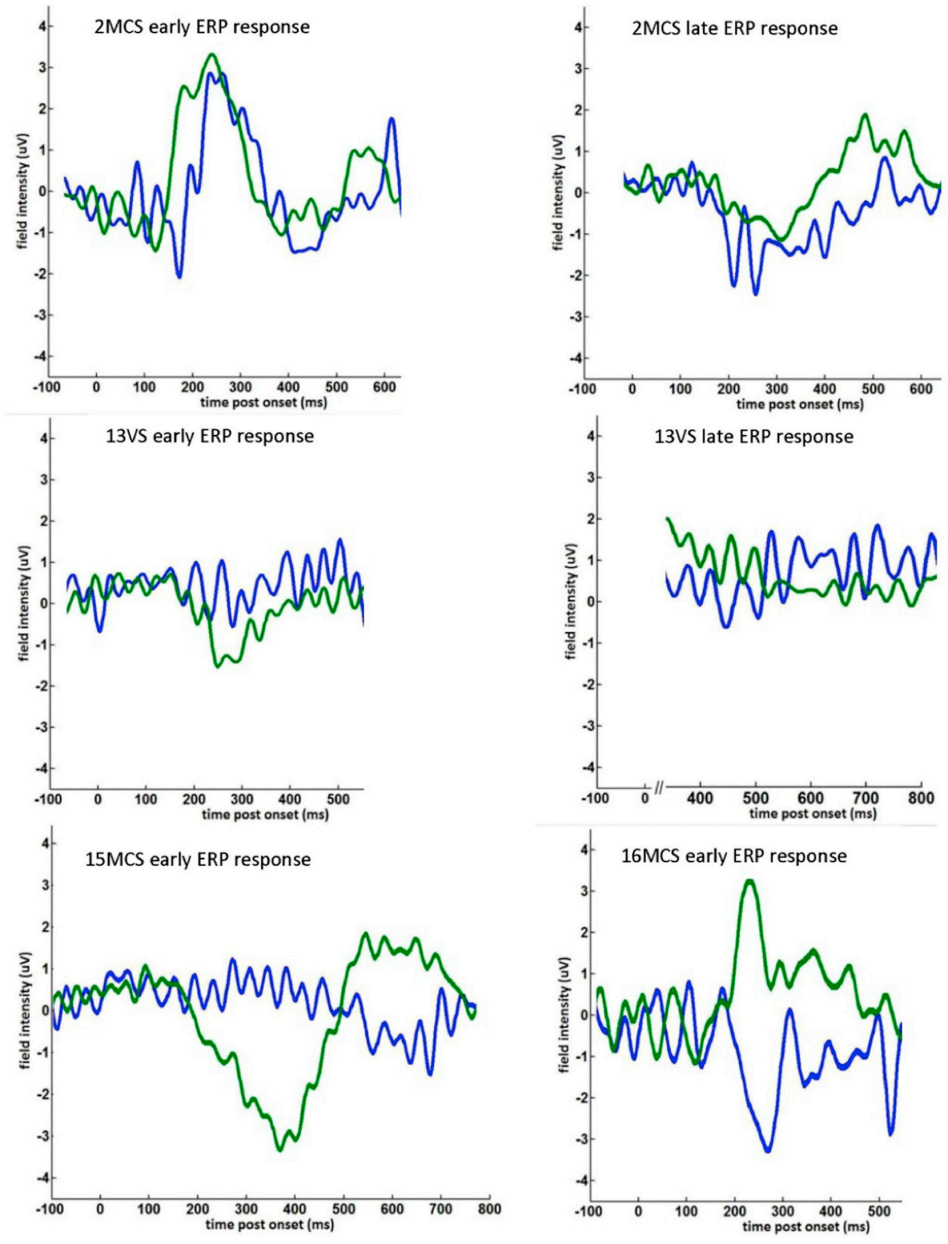
Represents the Event Related Response to own name (blue line) and other names (green line) in individual patients.

The data was analysed using the Statistical Parametric Software (*SPM 8*, *the Welcome Trust Centre for Neuroimaging*, *UCL*, *London*, *UK)*. The EEG data were converted from the *cnt format to the readable by SPM8 run using Matlab (*Matlab version R2011a*, *Mathworks*, *Natick MA*). The converted EEG data was subjected to a high-pass filter of 1 Hz to eliminate slow drift correction. Next eye artefact correction according to a script written within SPM8 (Berg eye movement correction) was performed. At this stage 2 out of 12 healthy subjects' and 1 out of 16 pDOC data sets were excluded from the further analysis due to excessive eye movement artefacts. Subsequently three epochs were created for each of the three conditions: SON, other names and reversed names with the epoch beginning 100 ms before the stimulus onset and ending 1000 ms later. The data were then low pass filtered at 30 Hz to eliminate muscle artefact and the EEG gamma frequency band. Subsequently 3D images were created for each trial, to represent changes of the scalp recorded potentials in scalp space (two dimensions, X and Y) over the peri-stimulus time (z dimension). (Litvak et al., 2011). A single image per trial contained information about the electrical signal in μV. For the controls, the data was taken to a second level of analysis, where one- way-ANOVA with R-levels and F-test was performed. The contrast between responses to own name versus responses to other names was examined for statistical differences, since this contrast was deemed as the most indicative of the self-awareness. The results were assessed with an F test for differences of either polarity. The results were initially thresholded at *p* = 0.001 uncorrected with a family wise error correction (FWE) correction based on random field theory (Litvak et al., 2011). As the whole epoch lasted 1100 ms and we were not expecting ERP responses throughout this entire time, we used two time defined volumes of interest at 250–350 ms and 600–800 ms post-stimulus. These masks were applied within SPM as a small volume correction (effectively the whole of sensor-space over these two-time windows). Only those regions, that survived a FWE correction of *p* < 0.05 corrected for multiple comparisons within these windows are reported (Litvak et al., 2011). As F tests identify differences between two conditions but do not indicate the directionality of effect, we plotted out the data for each significant response in controls (as a group, see Fig. 3 and Table 1) and patients (individually, see Fig. 4, Fig. 5, and Table 3). This analysis is in accordance with that suggested by Rousselet et al. (Rousselet et al., 2016) as it corrects for multiple comparisons across both sensor space and peri-stimulus time. For illustrative purposes only, ERPs were averaged across electrodes (and for controls across subjects) to produce grand mean ERPs for Fig. 3, Fig. 5.

**Table 1:** The responses from the healthy controls (*n* = 10) showing the difference between responses to SON vs. other names. The result was considered as significant if the correction using familywise error (FWE) and false discovery rate (FDR) were <0.05; the mm × mm shows the spatial position and ms indicates the time since onset for the most significant response.^⁎^positions taken from (Koessler et al., 2009).

10 controls, cluster-level data				Peak voxel data						
Time window	p FEW corrected	F	Z|	mm (L-R)	mm (A-P)	ms	Cortical projection^⁎^	Mean ERP own name μV	Mean ERP others' names μV	EEG electrodes averaged for ERPs in Fig. 3
250–350 ms	0.003	25.58	3.77	0	2	307	Superior frontal midline	0.2	2.8	Fz FCz Cz CPz; F1 FC1 C1 Cp1; F2 FC2 C2 CP2
600–800 ms	0.012	49.22	4.67	−34	−36	703	Superior parietal left	−1.1	1.7	T7 C5; M1 TP7 CP5; P7 P5

## 3 Results

### 3.1 EEG responses to SON in control population

The F test differences between responses to the SON and other names averaged for 10 controls were calculated. The table indicates all significant responses within the two-time windows of 250 to 350 ms and 600 to 800 ms post stimuli and the graphical presentation of sensor space*time (Fig. 3 and Table 1). Note that the response to others' names was greater than own name centrally at ~300 ms (positive polarity), while the later, left-lateralized response at ~700 ms was of a negative polarity for own name.

### 3.2 Study population characteristics

Sixteen patients with pDOC were included in the study (six female, mean age was 46 years, SD 11), with the following aetiologies: intracerebral haemorrhage (*n* = 6), anoxic brain injury (*n* = 5), traumatic brain injury (*n* = 4) and tuberculosis meningitis (*n* = 1). The patients had been in pDOC for 17.31 months on average (Table 2).

**Table 2:** Demographic data and characteristics of the study population. VS/UWS-Vegetative State/Unresponsive Wakefulness State, MCS-minimally conscious state, pDOC prolonged Disorders, of Consciousness, ^⁎^all included patients were diagnosed using The Sensory Modality Assessment and Rehabilitation Technique (SMART).

Patient's diagnosis^⁎^	Gender	Age in yrs.	Time since onset in months	Aetiology	Resting state EEG
1 MCS	F	18	4.7	Anoxic brain injury	Low amplitude without distinguishable features
2 MCS	F	61	55.1	Right frontal lobe bleed	Polymorphic activity at 2–2.5 Hz, small amount of beta activity at 16-18 Hz
3 MCS	M	55	9.1	Large intracerebral bleed	Diffuse polymorphic at 1.5–3.0 Hz, with low amplitude at 5–6 Hz superimposed, small amount of 18–20 Hz frontal bilaterally
4 VS/UWS	M	45	5.4	Anoxic brain injury post cardiac arrest	Low amplitude and without distinguishable features
5 MCS	M	68	4.0	Grade V SAH due to aneurysm left ACM	Diffuse activity at 1.0–1.5 Hz, rhythm at 8.5 Hz superimposed over post central regions
6 MCS	M	46	4.7	Extensive fronto-temporal left haemorrhage	Suppressed posterior rhythm, frontal derivation 4–6 Hz rhythm
7 MCS	M	38	9.1	Left fronto-temporo-parietal contusions following assault	Asymmetrical R > L, activity 5–7 Hz
8 MCS	F	30	80.9	Petechial haemorrhage following road traffic accident	Asymmetrical, diffuse activity at 1.5–3 Hz, occasional activity at 6 Hz central
9 MCS	F	37	1.8	Bilateral intracerebral bleed	Well- formed posterior rhythm 4–6 Hz
10 VS/UWS	M	24	6.4	Hydrocephalus following TB meningitis	Slow activity at 1–3 Hz, occasionally over right centro-temporal regions 4.0–4.5 Hz
11 VS/UWS	M	20	13.6	Diffuse axonal injury following road traffic accident	Diffuse polymorphic activity at 1.5–3.0 Hz, with low amplitude at 5–6 Hz
12 VS/UWS	M	51	40.4	Right temporo-parietal bleed	Over both hemispheres activity at 5–6 Hz, focal activity at 4–2.5 Hz over the right superior frontal region
13 VS/UWS	F	62	5.0	Anoxic brain injury post cardiac arrest	Symmetrical, low voltage activity mainly 3–4 Hz
14 MCS	M	52	6.4	Left parietal haemorrhage following road traffic accident	Activity at 5–6 Hz over central
15 MCS	F	31	26.0	Anoxic brain injury following cardiac arrest	Low amplitude activity at 2–4 Hz occasional activity at 7 Hz central
16 MCS	M	53	4.4	Anoxic brain injury following cardiac arrest	Occipital rhythm diminished, frontal 7–10 Hz

Our inclusion criterion for hearing ability was that each patient had at least unilateral positive brainstem auditory potentials. The BAEPs values were delayed (Supplement material Table 4) in 10 out of 16 prolonged DOC subjects with the mean time for inter-peak I to III of 2.55 ms, the reference value for normal subjects is 2.2 ms (Tusa et al., 1994). A similar delay was observed for the interpeak III-V, which in the pDOC patients was 2.1 ms, while the reference is 1.93 ms (Tusa et al., 1994), the interpeak I to V was only slightly delayed in the study population to 4.44 ms vs. 4.13 ms as a reference value (Tusa et al., 1994).

### 3.3 EEG responses to SON in pDOC patients

At the group level there were no significant differences between pDOC patients' responses to SON and other names. At the single subject level, however, we identified 4 pDOC patients (of the 15), who demonstrated significant responses between their own name vs. others' names. Two of these patients (numbered as: 2MCS and 16MCS) had responses very similar to controls in both timing and the positive polarity of the ERPs, while the other two patients (13VS/UWS and 15MCS) had statistically significant effects in the same time windows, but the direction of the effect was of the opposite polarity to controls (negative ERP to others' names) (Fig. 4, Fig. 5, Table 3).

**Table 3:** The responses from the four patients with the pDOC showing the difference between responses to SON vs. other names. The mm × mm shows the spatial position in MNI coordinates NB: −x value = left; −y value = behind the anterior commissure and ms indicates the time since stimulus onset for the most significant response. ^⁎^according to (Koessler et al., 2009).

Individual patient analyses cluster level data							Peak voxel data			
Subject number	p FEW corrected	F	Z|	mm (L-R)	mm (A-P)	ms	Cortical projection^⁎^	Mean ERP own name μV	Mean ERP others' names μV	EEG electrodes averaged for ERPs in Fig. 5
2MCS	<0.001	34.33	5.59	-4	-8	180	Inferior parietal midline	−2.8	2.8	POz PO4 PO6 PO8; PO3 PO5 PO7; O1 Oz O2
2MCS	0.013	25.09	4.78	−60	−52	471	Middle temporal left	−0.5	2.7	T7 C5; M1 TP7 CP5; P7 P5
13VS/UWS	0.021	20.46	4.30	4	−36	267	Pre-central /superior frontal midline	1.2	−2.1	Cz CPz
13VS/UWS	0.001	29.14	5.15	26	29	625	Superior frontal right	−5.3	0.6	AF4; F6 F8 F4
15MCS	0.065	16.19	3.80	13	−19	350	Post-central midline	0.5	−4.1	Fz FCz Cz CPz; F1 FC1 C1 Cp1; F2 FC2 C2 CP2
15MCS	0.016	21.02	4.36	4	−9	617	Post-central midline	−1.8	2.5	Fz FCz Cz CPz; F1 FC1 C1 Cp1; F2 FC2 C2 CP2
16MCS	0.050	22.14	4.48	−13	−62	268	Middle/inferior temporal left	−5.9	4.3	POz PO4 PO6 PO8; PO3 PO5 PO7; O1 Oz O2

## 4 Discussion

Our primary aim was to identify brain responses to subject's own name (SON) and others' names. We accomplished this by embedding SON stimuli in blocks of either others' names or reversed names to be sure that ERPs were in response to transient changes in the acoustic signal rather than fluctuations in brain state that may confound blocked experimental trials. We chose the contrast SON vs. others' names as these were the most similar conditions and differed in semantic salience. While the reversed names were part of the experimental paradigm, we did not contrast these trials directly with SON trials because any ERP differences could have been driven by either semantic or phonotactics differences. We did however utilize the SON trials that were embedded in the reversed name blocks. For the 10 control subjects we identified two main positive responses in keeping with previous studies: A) an early polarity positive response around 300 ms, which is typically maximal in the frontal or central midline electrodes (Berlad and Pratt, 1995); B) a later, also polarity positive, ERP between 600 and 800 ms, also known as a “late slow wave” with left parietal topology (Holeckova et al., 2006). The early response is deemed to reflect cognitive functions such as stimulus recognition and working memory updating (Coles et al., 1988), (Polich, 1987), while the late response is believed to occur during recollection and retrieval processes (Holeckova et al., 2006). The topology of these two responses in our control subjects was consistent with the earlier studies cited above i.e. central for the early response and left-lateralized (parietal) for the later response. The magnitude and polarity of the effect (with others' names having a greater ERP response than SON) at 300 ms is consistent with other passive paradigms (where subjects are asked to not attend specifically to the auditory stimuli (Hauger et al., 2015)). We chose a passive paradigm for the controls as we had no way of ascertaining whether patients would comply with a more active paradigm (e.g.: one where they are asked to count the number of times that they hear their own name).

In our patient group, 4/16 (25%) had statistically significant responses to SON vs others' names. Two had a very similar topology to controls while two had an opposite polarity in response to others' names. Two pDOC patients had significant response in both time windows, while one subject only had an early response. The final responder had two peaks occurring a little earlier than the controls. The topology (given that these patients had all suffered some form of global brain injury) was reasonably consistent with the control data, with most patients having a maximal response centrally for the early component (although one was quite posterior e.g.: 2MCS) and left-lateralized topography for the later response (2/3). We focused specifically on the difference in response to subject's own name and the response to other first names; because we think this contrast is the most sensitive to detecting recognition of personalized information.

Our study extends, those performed previously studies in this field through focussing on a salient contrast i.e. that between SON and others names. Fisher et al. (Fischer et al., 2008) assessed comatose rather that pDOC patients and found in 21 out of 51 a positive ERP to subject's own name at the latency of 602 ms, 671 ms and 722 ms at Fz, Cz and Pz respectively. Others showed that pDOC patients had stronger ERP response to own name uttered by familiar voice (Holeckova et al., 2006) (Del Giudice et al., 2016). Additionally, it was shown that pDOC patients in active condition such as counting down the SONs evoked stronger response then in unattended (passive) condition (Schnakers et al., 2015) (Hauger et al., 2015). Furthermore, a strong response to a SON in pDOC patients was reported if a SON was contrasted against a meaningless sound (Demertzi et al., 2008).

Previously, Perrin et al. (Perrin et al., 2006) and Schnakers et al. (Schnakers et al., 2008) showed that pDOC patients developed a delayed P300 response to SON, for instance, in VS patients the P300 was at 762 ms, in MCS at 711 ms, respectively.

Significant differences between brain responses to own versus others' names are unlikely to be due to any low-level auditory or even phonemic factors, but more plausibly to higher-level (auditory object) (Snyder et al., 2012) detection, or to the personal and emotional salience associated with hearing one's own name; hence, the positive results we observed in a sub-group of patients may be an index of a certain level of self-awareness. In our study, the VS/UWS patient who demonstrated consistent an ERP response to his/her own name was diagnosed using the SMART assessment, and was not thought to have any behavioural responses indicating self-awareness, or awareness of the environment. The clinical EEG of this patient was dominated by delta rhythm, yet they had normal BAEPs and a significant ERP (albeit of the opposite polarity to controls) for others' names vs SON. This finding is supportive of previous studies, strongly suggesting that a minority of VS/UWS patients have islands of preserved cognitive function (Formisano et al., 2013).

Various psychological experiments have shown that hearing one's own name can lead to an increase in attention. For instance, by using shadowing procedures or distractions, Howarth and Ellis (Howarth and Ellis, 1961) showed that the auditory threshold for perceiving one's own name was lower than for hearing other names. Others have suggested that there is a connection between one's name, personal identity, memory and attention (Dion, 1983). The finding of a smaller ERP to the subject's own name is consistent with the predictive coding account of brain function that postulates that familiar stimuli are recognised more efficiently by the brain with associated error signals (a mismatch between expectation and sensory information) being quashed more quickly and efficiently than those from unexpected or unpredictable stimuli (such as other peoples' names) (Garrido et al., 2009).

The finding of a negative polarity response to others' names in two of the patients (13VS at 267 = −2.1 mV; 15MCS at 350 = −4.1 mV, see Fig. 5 and Table 3) was surprising and difficult to interpret, although a similar result is reported by Schnakers et al. (Schnakers et al., 2008) for DOC patients when listening passively to others' names (see Fig. 5) with a negative peaking at around 400 ms = −2.5 mV. Perhaps, in these subjects, others' names are eliciting a form of MMN compared to own name (in our paradigm others' names were never interspersed with reversed names, as SON stimuli were).

Recently has it been proposed that proper assessment of brain function in pDOC patients requires person specific and person relevant stimuli. These stimuli, may be not only as mentioned above an auditory (SON); but can be visual, where pictures of one's own face are contrasted with unfamiliar faces (Laureys et al., 2007). Clinic studies further suggest that person-relevant stimuli may be best for bedside and behavioural assessment. Cheng et al. (Cheng et al., 2013) showed in eighty six VS/UWS subjects that using the patient's own name as opposed to a meaningless loud sound (i.e., ringing a bell), was more effective in evoking a localisation-to-sound response (i.e., turning eyes/head towards the sound source). Others have also suggested using personally meaningful stimuli for brain function assessment in pDOC patients, for instance, by using pre-injury personally relevant stimuli, such as patients' favourite music or similar (Perrin et al., 2015).

Nevertheless, the sensitivity of the use of EEG for detection of awareness is limited. It is known that some of the MCS patients or even healthy subjects do not respond on the event related paradigms (Cruse et al., 2012; Schnakers et al., 2015). In our study only 3 out of 10 MCS patients responded. A multi-dimensional approach for an EEG assessment of brain function in pDOC patient using SON stimuli amongst others such as temporal attention, spatial attention, motor planning and detection of spatial incongruence may better access awareness in pDOC patients who are functionally locked in (Sergent et al., 2017). Recently, it was shown that the low-frequency power, EEG complexity, and information exchange derived from the EEG were deemed as reliable features of consciousness in patients with disorders of consciousness (King et al., 2013) King and others (Sitt et al., 2014) suggested using EEG to assess an information sharing and signal propagation across distant sites of cortex, which can distinguish patients with various degree of consciousness impairment. The authors showed that information sharing was significantly lower in the VS/UWS patients regardless of aetiology of brain injury and time since onset. Not only EEG but also other methods such the transcranial magnetic stimulation technique and a Perturbational Complexity Index enables stratification patients into groups of VS/UWS versus MCS with high specificity and sensitivity (Casarotto et al., 2016).

An important limitation of the present study is that only 25% patients responded to a name paradigm and spatial localization of the responses was inconsistent within the subjects. However, this may be partially explained by the fact that we aimed to find the difference in brain responses to own name and other names. The EEG responses to this contrast have been localized within the superior medial frontal, temporal and parietal cortices that can be activated during self-referred stimuli (Knyazev, 2013).

Another limitation of this study is that EEG signal was not reconstructed to assess cortical generators of scalp potentials, as was performed by Friston et al. (Park and Friston, 2013), who shown that cortical processes implies a fast and effective dialogue between differentiated cortical areas. Salient stimuli, such as patient's own name can cause activation of subcortical structures, for instance, locus coeruleus. This would be, however, considered only as a preparation of cortical circuits for high-level cognitive processes, rather the actual cognitive processes. Hypothetically, a response in VS/UWS patients can be just caused the activation of subcortical structures, as the locus coeruleus, which prepare cortical circuits for high-level cognitive processes (Sara and Bouret, 2012). Hence, the fMRI technique is superior because of spacial localization of the BOLD signal. A fMRI study showed widespread activation to the SON of the posterior network: the middle temporal cortex, left superior temporal cortex, middle occipital gyrus and cuneus as well in frontal cortex (Carmody and Lewis, 2006). The feasibility of the use of fMRI and SON stimuli for the outcome prediction in TBI VS/UWS patients was shown by Wang et al. (Wang et al., 2015). Since both techniques are complementary, the use of both the fMRI and EEG could help to determine the sensitivity and specificity of EEG.

## 5 Conclusion

The results from our study suggest that names can be powerful stimuli for brain function assessment in pDOC patients, especially, if the response to SON is contrasted to a response to any other name. One out of five VS/UWS patients responded to this stimulus, indicating at least partial self-awareness. This study shows that EEG can be a robust technique for brain function assessment and be complementary with a gold standard bedside assessment.

The following is the supplementary data related to this article.

Table 4 Brainstem auditory evoked potentials (BAEP) responses for right and left side, the wave's latency is expressed in millisecond, inclusion criteria was to have at least unilateral positive BAEP. VS/UWS-vegetative state/Unresponsive Wakefulness State, MCS-minimally conscious state, pDOC prolonged Disorders, of Consciousness, * pDOC patients with the ERP response to subject’s own name, α pDOC patients with normal BAEP, defined as at least unilateral interpeak I to III is 2.1 ms and III-V −1.93 ms. Table 4

## Conflict of interest statement

The Authors declare that there is no conflict of interest.
