# Supplementary material for: Patients with a severe prolonged Disorder of Consciousness can show classical EEG responses to their own name compared with others' names
Source: Neuroimage Clin. 2018 Apr 30;19:311–9. doi: 10.1016/j.nicl.2018.04.027 (PMC6044184; doi:10.1016/j.nicl.2018.04.027)
Supplement: Table 4 — Brainstem auditory evoked potentials (BAEP) responses for right and left side, the wave's latency is expressed in millisecond, inclusion criteria was to have at least unilateral positive BAEP. VS/UWS-vegetative state/Unresponsive Wakefulness State, MCS-minimally conscious state, pDOC prolonged Disorders, of Consciousness, * pDOC patients with the ERP response to subject’s own name, α pDOC patients with normal BAEP, defined as at least unilateral interpeak I to III is 2.1 ms and III-V −1.93 ms. [file mmc1.docx]

|  | **Right ear** | | | | | | **Left ear** | | | |  |
| --- | --- | --- | --- | --- | --- | --- | --- | --- | --- | --- | --- |
| Patient’s number | I wave latency | II | III | IV | V | I | | II | III | IV | V |
| 1 MCS | 1.5 | 2.5 | 3.5 | 4.8 | 5.5 | 1.4 | | 2.7 | 3.5 | 4.8 | 5.6 |
| 2 MCS*α | 1.8 | 3.0 | 3.8 | 4.9 | 5.5 | 1.9 | | 3.0 | 3.9 | 4.6 | 5.5 |
| 3 MCS | 1.8 | 3.2 | 4.3 | 5.5 | 6.7 | absent | | absent | absent | absent | absent |
| 4 VS/UWS | 1.7 | 2.4 | 5.0 | 7.6 | 8.2 | 1.6 | | 2.3 | 3.7 | 5.9 | 6.4 |
| 5 MCSα | absent | absent | absent | absent | absent | 1.9 | | 2.7 | 3.9 | 4.9 | 5.4 |
| 6 MCS | 1.6 | 2.6 | 4.0 | 5.1 | 5.6 | 1.6 | | 3.2 | 3.7 | 5.3 | 6.1 |
| 7 MCS | 1.5 | 2.5 | 4.0 | 5.7 | 6.8 | absent | | absent | absent | absent | absent |
| 8 MCSα | 1.4 | 2.4 | 3.5 | 4.6 | 5.2 | 1.9 | | 2.7 | 3.7 | 4.7 | 5.3 |
| 9 MCSα | 1.6 | 2.8 | 3.9 | 5.2 | 6.0 | 1.5 | | 2.8 | 3.8 | 5.0 | 5.5 |
| 10 VS/UWS | 1.6 | 3.7 | 5.1 | 6.0 | 7.9 | 1.6 | | 2.8 | 3.6 | 5.0 | 5.7 |
| 11 VS/UWS | 2.1 | 4.2 | 5.8 | 7.0 | 8.2 | 1.8 | | absent | 3.9 | absent | absent |
| 12 VS/UWS | absent | absent | absent | absent | absent | 1.5 | | 2.7 | 3.9 | 6.0 | 7.1 |
| 13 VS/UWS*α | 1.5 | 2.3 | 3.9 | 4.9 | 5.5 | 1.5 | | 2.3 | 3.7 | 4.9 | 5.5 |
| 14 MCS | 1.7 | 3.3 | 4.2 | 5.3 | 5.7 | 1.6 | | 3.0 | 4.0 | 5.3 | 6.0 |
| 15 MCS*α | 1.4 | 2.6 | 3.5 | 4.5 | 5.3 | 1.6 | | 2.9 | 3.9 | 5.0 | 5.9 |
| 16 MCS*α | 1.4 | 3.0 | 4.1 | 5.1 | 5.8 | absent | | absent | absent | absent | absent |

***Table 4 Brainstem Auditory Evoked Potentials (BAEP) responses for right and left side, the wave’s latency is expressed in millisecond, inclusion criteria was to have at least unilateral positive BAEP. VS/UWS-vegetative state/Unresponsive Wakefulness State, MCS-minimally conscious state, pDOC prolonged Disorders, of Consciousness, * pDOC patients with the ERP response to subject’s own name, α pDOC patients with normal BAEP, defined as at least unilateral interpeak I to III is 2.1ms and III-V -1.93ms***
